# Serotype distribution and antimicrobial susceptibility of *Streptococcus pneumoniae* isolates cultured from Japanese adult patients with community-acquired pneumonia in Goto City, Japan

**DOI:** 10.3389/fmicb.2024.1458307

**Published:** 2024-09-24

**Authors:** Taiga Miyazaki, Mark van der Linden, Katsuji Hirano, Takahiro Maeda, Shigeru Kohno, Elisa N. Gonzalez, Pingping Zhang, Raul E. Isturiz, Sharon L. Gray, Lindsay R. Grant, Michael W. Pride, Bradford D. Gessner, Luis Jodar, Adriano G. Arguedas

**Affiliations:** ^1^Nagasaki University, Nagasaki, Japan; ^2^Division of Respirology, Rheumatology, Infectious Diseases, and Neurology, Department of Internal Medicine, Faculty of Medicine, University of Miyazaki, Miyazaki, Japan; ^3^German Reference Laboratory for Streptococci, Department of Medical Microbiology, University Hospital RWTH, Aachen, Germany; ^4^National Center for Global Health and Medicine, Tokyo, Japan; ^5^Pfizer Inc., Collegeville, PA, United States; ^6^Pfizer Inc., Pearl RiverNew York, NY, United States

**Keywords:** antimicrobial susceptibility, community-acquired pneumonia, PCV, pneumococcal vaccination, *Streptococcus pneumoniae*

## Abstract

*Streptococcus pneumoniae* is an important cause of community-acquired pneumonia (CAP) in Japan. Here, we report the serotype distribution and antimicrobial susceptibility of cultured pneumococcal isolates from Japanese adults aged ≥18 years with CAP. This was a prospective, population-based, active surveillance study conducted in Goto City, Japan from December 2015 to November 2020. Pneumococcal isolates from sterile sites (blood and pleural fluid) and non-sterile sites (sputum and bronchoalveolar lavage) were cultured as part of the standard of care. *S. pneumoniae* were serotyped using the Quellung reaction. Antimicrobial susceptibility was tested using microdilution and interpreted according to the Clinical and Laboratory Standards Institute criteria. Isolates resistant to erythromycin were phenotyped using the triple-risk test and genotyped by polymerase chain reaction. A total of 156 pneumococcal isolates were collected (138 from sputum, 15 from blood, and 3 from bronchoalveolar lavage) from 1992 patients. Of these, 142 were non-duplicate isolates from unique patients and were included in the analyses. Serotypes contained within the 13-valent pneumococcal conjugate vaccine (PCV13) (including 6C), PCV15 (including 6C), and PCV20 (including 6C and 15C) were detected in 39 (27%), 45 (32%), and 80 (56%) of 142 isolates, respectively. The most common serotypes were 35B (12%), 11A (11%), and 3 (11%). Multidrug resistance (MDR) was detected in 96/142 (68%) isolates. Of the 96 MDR isolates, 31, 32, and 59% were PCV13, PCV15, and PCV20 serotypes, respectively; the most common MDR serotypes were 35B (16%), 6C, 10A, and 15A (9% each), and 3 and 11A (8% each). A total of 119 isolates were resistant to macrolides; 41 (35%) had an M phenotype, 53 (45%) had an iMcLS phenotype, and 25 (21%) had a cMLS phenotype. In conclusion, pneumococcal serotypes 35B, 11A and 3 were most frequently associated with pneumonia and antimicrobial resistance was common among pneumococcal isolates from adults with CAP in Goto City, Japan. Implementing higher-valency PCVs May help reduce vaccine-type CAP among Japanese adults.

## 1 Introduction

Community-acquired pneumonia (CAP) is an important cause of morbidity and mortality. In Japan, pneumonia was ranked as the fifth leading cause of death in 2019 (Vital Statistics of Japan, 2019). As older adults are at high risk of pneumonia, the increasing burden of pneumonia among Japan’s aging population has a significant impact on the healthcare system (Glick et al., 2021).

*Streptococcus pneumoniae* is one of the most common bacterial causes of CAP (Johansson et al., 2010). However, with the introduction of pneumococcal conjugate vaccines (PCVs) for pediatric populations globally, there has been a substantial reduction of pneumococcal disease caused by vaccine serotypes both in children and unvaccinated individuals through herd protection (Musher et al., 2022). In Japan, a 7-valent PCV (PCV7) was incorporated into the infant National Immunization Program (NIP) in April 2013, replaced by a 13-valent PCV (PCV13) in November 2013 (Yanagihara et al., 2021). Both PCV13 and a 23-valent pneumococcal polysaccharide vaccine (PPSV23) are available for adult use. PCV13 was approved against pneumococcal disease for adults aged ≥65 years on a voluntary basis in 2014 and in 2020 the indication was expanded to include individuals 6 to 64 years of age with certain medical conditions. PPSV23 has been included in the adult NIP since 2014, and it is recommended for adults aged ≥65 years and adults aged 60–64 years with certain medical conditions (Yanagihara et al., 2021).

With the widespread use of PCVs in infants, pneumococcal epidemiology has shifted, and several studies have documented the rise of non-vaccine serotypes in respiratory carriage and disease, a process known as serotype replacement or the result of not having other antigens in previous formulations. To address serotype replacement, vaccine manufacturers have developed higher valency PCVs to expand serotype coverage. In 2021, two new PCVs, a 15-valent PCV (PCV15) and a 20-valent PCV (PCV20), were licensed by the Food and Drug Administration in adults aged ≥18 years old. The US Advisory Committee for Immunization Practices recommendations specify the use of either PCV20 alone or PCV15 in series with PPSV23 for all adults aged ≥65 years and for adults aged 19–64 years with certain underlying medical conditions or other risk factors who have not received a PCV or whose vaccination history is unknown (Kobayashi et al., 2023). Both, vaccines—PCV15 and PCV20—were also approved in Europe by the European Medicines Agency for prevention of invasive disease and pneumonia in individuals ≥18 years of age (European Medicines Agency, 2023).

In Japan, PCV15 is recommended as an optional sequential vaccine with PPSV23 for all adults aged ≥65 years and individuals aged 18–64 years who are at high risk of pneumococcal disease but it is not included in the adult NIP (Japanese Association for Infectious Diseases, 2023). Currently, PCV20 is being evaluated in clinical trials for individuals aged 18–49 years old and in individuals aged ≥65 years (Hoshi et al., 2022).

Although there have been studies assessing the burden of pneumococcal disease in Japan, most of the studies have focused on invasive pneumococcal disease (IPD) (Igarashi et al., 2022) and data on CAP epidemiology among Japanese adults are limited except for the current study in Goto City (Miyazaki et al., 2023), previous CAP studies were done before the introduction of PPSV23 into the adult NIP in October 2014 (Morimoto et al., 2015).

To address the research gap, we conducted the Goto Epidemiology Study, a prospective, population-based, CAP surveillance study in Goto City, Japan, between December 2015 and November 2020. This is the third of three companion papers from the Goto Epidemiology Study (Glick et al., 2021; Miyazaki et al., 2023). Incidence of CAP and pneumococcal CAP, and serotype distribution of non-bacteremic CAP from the same study were summarized in a previous publication (Miyazaki et al., 2023). The aim of this study is to report the serotype distribution and antimicrobial susceptibility of cultured *S. pneumoniae* isolates obtained from adult patients with CAP.

## 2 Materials and methods

### 2.1 Patients and clinical isolates

The study included all healthcare facilities in Goto City. Patients aged ≥18 years who presented to a healthcare facility between December 2015 and November 2020 with signs, symptoms, and radiographic evidence of pneumonia were screened for inclusion. Detailed patient inclusion/exclusion criteria, demographics, disease severity, healthy status, use of antimicrobials, hospitalization and disease outcomes were described in a previous publication (Miyazaki et al., 2023). Clinical specimens, including sputum and those from normally sterile sites, were collected from the patients as part of standard-of-care. Herein, we present the results on the serotype distribution and antimicrobial susceptibility from cultured isolates.

### 2.2 *Streptococcus pneumoniae* detection

Clinical isolates were initially processed at the Goto Central Hospital Laboratory for *S. pneumoniae* identification. Gram stain was not routinely performed in sputum samples. Isolates were cultured on blood agar plates with gentamycin. Colonies showing *α*-hemolysis were processed for *S. pneumoniae* and confirmed by optochin susceptibility and bile solubility testing. Optochin susceptibility testing was performed in a 5% CO_2_ atmosphere on sheep blood agar. Bile solubility testing was performed using a bacterial suspension in 1 mL 0.85% NaCl and adding four drops of 10% sodium deoxycholate (van der Linden et al., 2009). All *S. pneumoniae* isolates were then stored at −80°C in porous beads (Microbank; Pro-Lab Diagnostics, Richmond Hill, ON, Canada) until shipped on dry ice to the German Reference Laboratory for Streptococci for further identification, serotyping and antimicrobial susceptibility testing.

### 2.3 Serotyping

As previously described, serotyping of *S. pneumoniae* was performed using the Neufeld-Quellung reaction with antisera from Statens Serum Institute, Copenhagen, Denmark (Imöhl et al., 2021). PCV13 serotypes include 1, 3, 4, 5, 6A, 6B, 7F, 9V, 14, 18C, 19A, 19F, and 23F. PCV15 serotypes include 22F, 33F, in addition to PCV13 serotypes. PCV20 serotypes include 8, 10A, 11A, 12F, 15B, in addition to PCV15 serotypes. Considering 6C and 15C are non-PCV13 and non-PCV20 serotypes but cross-reacting with 6A and 15B respectively, PCV13 (including 6C), PCV15 (including 6C), and PCV20 (including 6C and 15C) serotypes were summarized in this paper. PCV and/or each serotype distribution by culture were estimated overall and for the 18–64 and ≥65 years age groups. When more than one pneumococcal isolate of the same serotype was available from the same episode for an individual, only one was counted in the analysis. There were no cases in which different pneumococcal serotypes were cultured from the same patient but from a different source. The number of isolates with unique serotype was used as the denominator for percentage calculation (*n* = 142). For multidrug resistance (MDR) of PCV, the number of total isolates with multidrug resistant pneumococci was used as the denominator for percentage calculation (*n* = 96).

### 2.4 Macrolide resistance phenotypes and genotypes

Macrolide resistance was investigated using erythromycin, and resistant isolates were phenotyped using the triple-disk test (erythromycin and clindamycin plus josamycin/rokitamycin) as described by Giovanetti and colleagues (Giovanetti et al., 1999), and genotyped by PCR as described previously (Bley et al., 2011). *Streptococcus pneumoniae* ATCC 49619^™^ was used as a control strain.

### 2.5 Antimicrobial susceptibility testing

Tested antimicrobial agents included amoxicillin, penicillin, cefotaxime, erythromycin, clindamycin, tetracycline, trimethoprim/sulfamethoxazole (TMP/SMX), levofloxacin, and vancomycin. Minimal inhibitory concentration (MIC) testing was performed using microdilution with Mueller-Hinton broth plus 5% lysed horse blood. The final inoculum concentration was 0.5 × 10^6^ colony forming units/ml. The minimal inhibitory concentration breakpoints and interpretive categories (susceptible, intermediate, and resistant) used in this study for *S. pneumoniae* were based on values established in the Clinical and Laboratory Standards Institute (Clinical and Laboratory Standards Institute, 2024). For penicillin (parenteral) and amoxicillin, MIC ≤2 μg/mL was considered susceptible, MIC of 4 μg/mL intermediate and an MIC ≥8 μg/mL resistant; for penicillin (oral), MIC ≤0.06 μg/mL was considered susceptible, MIC of 0.12–1 μg/mL intermediate and an MIC ≥2 μg/mL resistant; for cefotaxime, an MIC ≤1 μg/mL was considered susceptible, MIC of 2 μg/mL intermediate, and an MIC ≥4 μg/mL resistant; for erythromycin and clindamycin, an MIC ≤0.25 μg/mL was considered susceptible, an MIC of 0.5 μg/mL intermediate and an MIC of ≥1 μg/mL resistant; for tetracycline, an MIC ≤1 μg/mL was considered susceptible, an MIC of 2 μg/mL intermediate and an MIC ≥4 μg/mL resistant; for TMP/SMX, an MIC of ≤0.5/9.5 μg/mL was considered susceptible, an MIC between 1/19 μg/mL and 2/38 μg/mL intermediate and an MIC ≥4/76 μg/mL resistant; for levofloxacin, an MIC ≤2 μg/mL was considered susceptible, an MIC of 4 μg/mL intermediate and for vancomycin, an MIC of <1 μg/mL was considered susceptible.

MIC_50_ was defined as the MIC of a given antimicrobial that inhibited growth of 50% of the isolates, while MIC_90_ was defined as the MIC of a given antimicrobial that inhibited growth of 90% of the isolates. MDR was defined as resistance to three or more antimicrobial classes (intermediate and resistant for penicillin, and resistant for other antimicrobials).

### 2.6 Statistical analysis

Descriptive statistical analysis was performed for categorical variables, which were described as frequencies and percentages. The distribution of pneumococcal serotypes between age groups was compared using Fisher’s exact test (GraphPad Prism version 10, GraphPad Software, Boston, MA). A *p* value of <0.05 was considered statistically significant.

## 3 Results

A total of 156 pneumococcal isolates were collected from 1992 patients, with 138 isolates from sputum, 15 from blood, and 3 from bronchoalveolar lavage. Of these, 142 isolates with unique serotype were included in the analysis: 55 isolates were from patients 18–64 years old and 87 isolates were from patients aged ≥65 years old (Supplementary Table S1).

### 3.1 Prevalence of serotypes from cultured pneumococcal isolates

**Figure 1:**
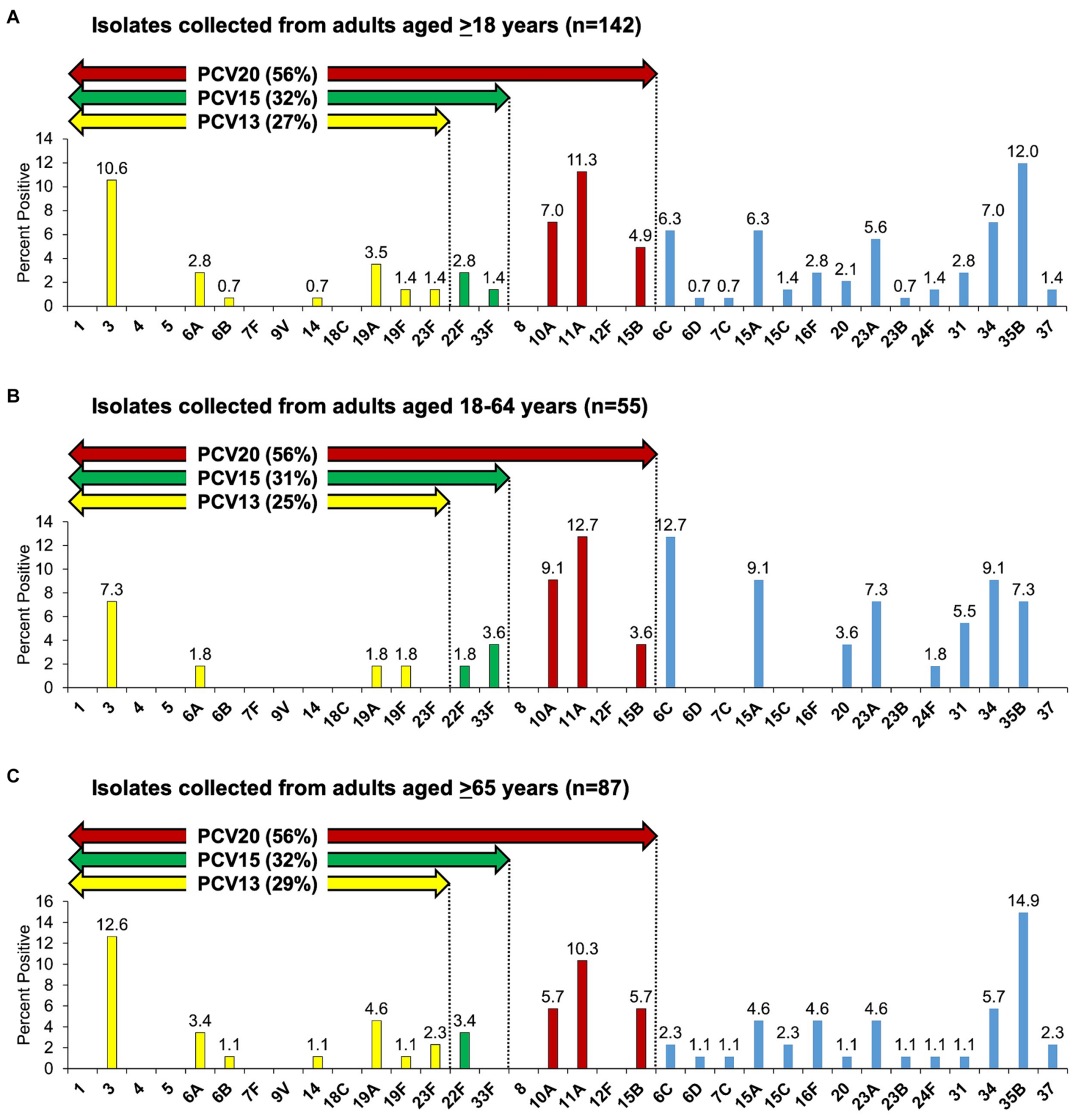
*Streptococcus pneumoniae* serotype distribution as detected by culture among adults with community-acquired pneumonia. **(A–C)** Serotypes 6C and 15C are non-PCV13 and non-PCV20 serotypes but cross-reacting with 6A and 15B, respectively, therefore PCV13 (including 6C), PCV15 (including 6C), and PCV20 (including 6C and 15C) serotypes are summarized.

Among all 142 isolates with unique serotype from patients aged ≥18 years, the most common serotypes were 35B (12.0%, 17/142), 11A (11.3%, 16/142), and 3 (10.6%, 15/142) (Figure 1A). Among 55 isolates with unique serotype from patients aged 18–64 years, the most common serotypes were 6C and 11A (12.7% each, 7/55), and 10A, 15A, and 34 (9.1% each, 5/55) (Figure 1B), whereas among 87 isolates with unique serotype from patients aged ≥65 years, the most common serotypes were 35B (14.9%, 13/87), 3 (12.6%, 11/87), and 11A (10.3%, 9/87) (Figure 1C).

Overall, PCV13 (including 6C), PCV15 (including 6C), and PCV20 (including 6C and 15C) serotypes were detected in 39 (27%, 39/142), 45 (32%, 45/142), and 80 (56%, 80/142) isolates, respectively (Figure 1A). When stratified by age, among 55 isolates collected from patients aged 18–64 years, PCV13 (including 6C), PCV15 (including 6C), and PCV20 (including 6C and 15C) serotypes represented 25% (14/55), 31% (17/55), and 56% (31/55) of the isolates, respectively (Figure 1B), while among 87 isolates collected from patients aged ≥65 years, PCV serotype coverage (in the same order) was 29% (25/87), 32% (28/87), and 56% (49/87) (Figure 1C). There was no statistical significance in the distribution of PCV13, PCV15, and PCV20 serotypes between the two age groups, 18–64 and ≥65 years of age.

### 3.2 Antimicrobial susceptibility of cultured pneumococcal isolates

Table 1 shows the antimicrobial MIC ranges, and MIC_50_ and MIC_90_ values. Among the 142 unique *S. pneumoniae* isolates, the proportions of isolates that were susceptible, intermediate, and resistant to the tested antibiotic were as follows: to amoxicillin, 100%, 0%, and 0%; to cefotaxime, 96.5%, 1.4%, and 2.1%; to clindamycin, 46.5%, 0%, and 53.5%; to erythromycin, 16.2%, 0%, and 83.8%; to levofloxacin, 100%, 0%, and 0%; to penicillin (oral), 61.3%, 28.2%, and 10.6%; to penicillin (parenteral), 97.9%, 2.1%, and 0%; to tetracycline, 19.7%, 2.8%, and 77.5%; to TMP/SMX, 81.7%, 12.7%, and 5.6%; to vancomycin, 100%, 0%, and 0%.

**Table 1:** Antimicrobial susceptibilities of 142 *Streptococcus pneumoniae* isolates obtained from adults with community-acquired pneumonia.

Antimicrobial	MIC range^a^	MIC_50_^b^	MIC_90_^c^
Amoxicillin	0.015–2	0.03	1
Penicillin	0.015–4	0.03	2
Cefotaxime	0.015–8	0.25	1
Clindamycin	0.12–256	64	256
Erythromycin	0.12–256	256	256
Tetracycline	0.12–128	32	64
Trimethoprim/Sulfamethoxazole	0.25/4.75–8/152	0.25/4.75	1/19
Levofloxacin	0.5–2	1	1
Vancomycin	0.5–0.5	0.5	0.5

a MIC, minimal inhibitory concentration values are in mg/L.

b MIC_50_, MIC that inhibited growth of 50% of the isolates.

c MIC_90_, MIC that inhibited growth of 90% of the isolates.

Among 119 macrolide resistant isolates, 41 (34.5%) had an M phenotype, 53 (44.5%) had an inducible macrolide but constitutive lincosamide and streptogramin B resistance (iMcLS) phenotype, and 25 (21%) had a constitutive macrolide, lincosamide, and streptogramin B resistance (cMLS) phenotype. All 41 isolates with an M phenotype carried a *mefE* gene, and 4 also had the *ermB* gene. All iMcLS isolates carried an *ermB* gene, with 11 also having a *mefE* gene. All cMLS isolates had an *ermB* gene, and 8 also carried *mefE*. The M phenotype isolates showed MICs ranging from 1–8 μg/mL, showing that in the 4 isolates also having an *ermB* gene, this gene was most probably not functional (Table 2).

**Table 2:** Phenotype and genotype among 119 macrolide-resistant-isolates of *Streptococcus pneumoniae* collected from adults with community-acquired pneumonia.

Phenotype	*n*	Genotype				
		*mefA*	*mefE*	*mefA* + *mefE*	*ermB*	*mefE* + *ermB*
M	41	0	37	0	0	4
iMcLS	53	0	0	0	42	11
cMLS	25	0	0	0	17	8
Total	119	0	37	0	59	23

iMcLS, inducible macrolide but constitutive lincosamide and streptogramin B resistance; cMLS, constitutive macrolide, lincosamide, and streptogramin B resistance.

**Figure 2:**
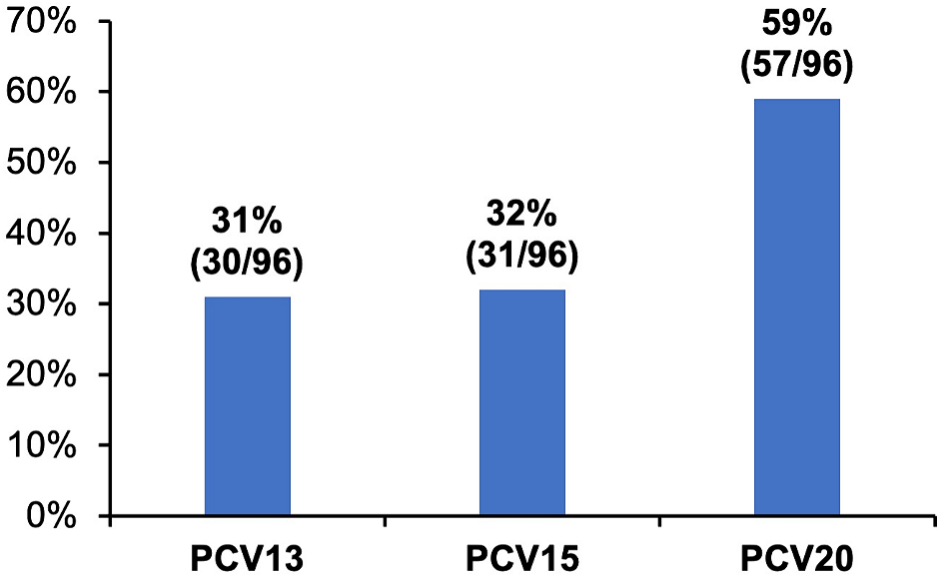
Proportions of vaccine-type serotypes of multidrug resistant *Streptococcus pneumoniae* isolates cultured from adults with community-acquired pneumonia. Multidrug resistance (MDR) was defined as resistance to three or more antimicrobial classes. Proportions of MDR isolates (*n* = 96) covered by different pneumococcal conjugate vaccines (PCV) are shown.

MDR was found in 96/142 (67.6%) isolates. Of MDR pneumococci, 31%, 32%, and 59% were associated with serotypes included in PCV13 (including 6C), PCV15 (including 6C), and PCV20 (including 6C and 15C), respectively (Figure 2); the most common MDR serotypes were 35B (16%), 6C, 10A and 15A (9%, each), and 3 and 11A (8%, each).

## 4 Discussion

In this 5-year CAP surveillance study in Goto City, Japan, we evaluated the serotype distribution and antimicrobial susceptibility of *S. pneumoniae* isolates cultured from adult patients with CAP between December 2015 and November 2020. A total of 142 unique pneumococcal isolates were cultured from 1992 patients and the most common serotypes were 35B (12%), 11A (11%), and 3 (11%). Among these isolates, 27%, 32%, and 56% were PCV13 (including serotype 6C), PCV15 (including 6C), and PCV20 (including 6C and 15C) serotypes, respectively. The isolates exhibited high macrolide resistance rate (84%) and MDR phenotypes (68%). Importantly, PCV serotype coverage was similar even for the MDR isolates; 31%, 32%, and 59% were PCV13, PCV15, and PCV20 serotypes, respectively.

Most of the pneumococcal strains were cultured from sputum samples (138 isolates). Although obtaining routine sputum samples for Gram stain and culture from patients with CAP remains controversial and is not routinely recommended in some settings (Metlay et al., 2019), sputum Gram staining and culture are recommended in Japan to identify causative microorganisms and select subsequent treatment strategies (Mikasa et al., 2016). Previous studies conducted among Japanese patients with CAP showed high specificity of Gram stain, which is therefore considered useful in guiding pathogen-targeted antibiotic empirical treatment (Fukuyama et al., 2014). The arguments for obtaining sputum samples to determine CAP etiology include the potential identification of resistant microorganisms, the possibility of targeted antimicrobial therapy, the detection of certain pathogens such as *Legionella pneumophila* and *Mycobacterium tuberculosis*, which may have public health implications, the option for treatment adjustments if patients do not respond to the initial empiric therapy, and the changing CAP epidemiology requiring ongoing evaluations (Metlay et al., 2019). Additionally, in a population like Japan, where PCVs are not recommended, upper respiratory tract cultures may provide evidence regarding the circulating pneumococcal serotypes, antimicrobial susceptibility and the potential impact that higher-valency PCVs may have in the community (Arguedas et al., 2020; Goldblatt et al., 2013).

Similar to the results reported from a recent multicenter study among Japanese patients with IPD (Yanagihara et al., 2021), most of the pneumococcal isolates in our study were susceptible to amoxicillin (100%), levofloxacin (100%), vancomycin (100%) and cefotaxime (96.5%), but highly resistant to erythromycin (83.8%). The percentages of penicillin-susceptible pneumococcal isolates in this study were also similar to those observed in the multicenter IPD study (61.3% versus 67.2%, respectively, for oral penicillin, and 97.9% versus 98.3%, respectively, for parenteral penicillin) (Yanagihara et al., 2021). Of the isolates cultured in this study, 18.3% of the strains were non-susceptible to TMP/SMX.

Moreover, among isolates in the current study, resistance rates were high for clindamycin (53.5%) and tetracycline (77.5%). Notably, most of the isolates that were resistant to erythromycin were also resistant to clindamycin (76 clindamycin-resistant isolates/119 erythromycin-resistant isolates, 64%). Also, we were able to document that the most common mechanism of resistance present in 68.9% of the erythromycin-resistant isolates involved modification due to a ribosomal methylase encoded by *erm(B)*, which confers high-level resistance to macrolides, lincosamides, and streptogramin B (MLS phenotype) (Del Grosso et al., 2007). The high prevalence of macrolide resistance is frequent among Japanese patients with pneumococcal infections (Yanagihara et al., 2021) and is usually associated with excessive use of macrolides (Pihlajamäki et al., 2003), particularly azalides with a long half-life like azithromycin (Dagan et al., 2009). Although we did not collect information regarding macrolide use in Goto City, one of the main reasons for the high macrolide resistance rate of *S. pneumoniae* in Japan compared to other countries, is the frequent use of small-dose long-term macrolide therapy for sino-bronchial syndromes such, as diffuse pan-bronchiolitis (Smith et al., 2020; Ide et al., 2023; Isozumi et al., 2007). In addition, among the 119 erythromycin-resistant isolates, 110 strains were non-susceptible to tetracycline (107 resistant and 3 intermediate). Tetracycline resistance is also frequently associated with erythromycin resistance worldwide (Doern et al., 2001; Seral et al., 2001; Marchese et al., 2000) and is predominantly due to ribosomal protection by the production of cytoplasmic proteins, making the interaction between tetracycline and the 30S ribosomal subunit difficult (Chopra and Roberts, 2001; Montanari et al., 2003).

Treatment guidelines for CAP differ across regions globally (Bender and Niederman, 2018). In Japan, due to the high prevalence of macrolide-resistant *S. pneumoniae* isolates and the possibility of infection caused by *Haemophilus influenzae*, and *Moraxella catarrhalis*, high-dose penicillin with a beta-lactamase inhibitor is recommended as the drug of choice for empiric therapy of CAP (Mikasa et al., 2016). In elderly patients and those with underlying lung diseases, a respiratory quinolone should be considered. For targeted therapy of *Mycoplasma pneumoniae* pneumonia, a macrolide or tetracycline is the first choice. The antimicrobial susceptibility data in this study support the use of high-dose oral amoxicillin as the first-line treatment for adult outpatients with pneumococcal CAP. This is consistent with the guidelines issued by the Japanese Association for Infectious Disease (JAID) and the Japanese Society of Chemotherapy (JSC) (JAID/JSC) for the treatment of respiratory infectious diseases in Japan regarding the high resistance rates observed against macrolides (Mikasa et al., 2016).

Our analysis showed that a substantial proportion of pneumococcal isolates were PCV serotypes, with roughly one-third due to PCV13 (27%) serotypes and more than half caused by PCV20 (56%) serotypes. Of the three most common serotypes detected from pneumococcal cultures among patients ≥18 years, serotypes 11A and 3 are included in PCV20 but serotype 35B is currently not included in any approved pneumococcal vaccine. Further, about two-thirds of MDR isolates were covered by PCV20 serotypes (59%). Recent surveillance studies conducted in Japan have shown an increase in infections caused by serotypes 35B and 15A (Ono et al., 2023; Ubukata et al., 2015; Nakano et al., 2020; Kawaguchiya et al., 2020) and in our study, these serotypes were among the most common serotypes detected in all patients aged ≥18 years (12 and 6.3%, respectively), particularly in adults ≥65 years (14.9 and 4.6% respectively) and furthermore, 16% of 35B isolates and 9% of 15A serotypes were MDR.

This study had a few limitations. First, individuals were recruited exclusively from a single community in Japan. The participants were primarily ≥65 years of age and were diagnosed with moderate to severe CAP at the time of enrollment. However, Goto City is located in the Sea of Japan, and the demographic characteristics of Goto City residents closely resemble those of Japan as a whole. Although the population aging rate (≥65 years) in Goto City (40.1% in 2021) was higher than that in Japan as a whole (29.2% in 2023), Goto City is epidemiologically similar to Japan as whole (Statistics Bureau of Japan, 2024). Therefore, the findings in this study are generalizable to Japan. Second, most of the isolates were obtained from sputum samples (88.5%) cultured without prior Gram stain. In addition, the pneumococcal vaccination status in the entire adult population in Goto City was unknown. Third, information was not available regarding antimicrobial usage in the community, which might have helped in explaining the high antimicrobial resistant rates against certain antimicrobials such as erythromycin.

In conclusion, there is a high level of antimicrobial resistance among pneumococcal isolates obtained from sputum and sterile sites among adults with CAP in Goto City, Japan. Despite a mature PCV13 pediatric immunization program in Japan since 2013, PCV13 serotypes continue to circulate among adults, illustrating the potential limitations of relying on pediatric immunization to achieve herd protection. Because PCV20 serotypes were detected in more than half of the pneumococcal isolates and approximately two thirds of multidrug resistant pneumococci, PCV20 use in adults could substantially reduce vaccine-type CAP burden in Japan. Results from the study highlight the importance of appropriate use of antibiotics to help reduce the emergence of resistant *S. pneumoniae* and the need of continuous surveillance to monitor changes in pneumococcal resistance patterns.

## Data Availability

The original contributions presented in the study are included in the article/Supplementary material, further inquiries can be directed to the corresponding author.
